# Structure of a Blinkin-BUBR1 Complex Reveals an Interaction Crucial for Kinetochore-Mitotic Checkpoint Regulation via an Unanticipated Binding Site

**DOI:** 10.1016/j.str.2011.09.017

**Published:** 2011-11-09

**Authors:** Victor M. Bolanos-Garcia, Tiziana Lischetti, Dijana Matak-Vinković, Ernesto Cota, Pete J. Simpson, Dimitri Y. Chirgadze, David R. Spring, Carol V. Robinson, Jakob Nilsson, Tom L. Blundell

**Affiliations:** 1 Department of Biochemistry, University of Cambridge, Cambridge CB2 1GA, UK; 2 The NNF Center for Protein Research, Faculty of Health Sciences, University of Copenhagen, Blegdamsvej 3B, DK-2200 Copenhagen, Denmark; 3 Department of Chemistry, University of Cambridge, Cambridge CB2 1EW, UK; 4 Department of Life Sciences, Imperial College London, London SW7 2AZ, UK; 5 Department of Chemistry, University of Oxford, Oxford OX1 3QZ, UK

## Abstract

The maintenance of genomic stability relies on the spindle assembly checkpoint (SAC), which ensures accurate chromosome segregation by delaying the onset of anaphase until all chromosomes are properly bioriented and attached to the mitotic spindle. BUB1 and BUBR1 kinases are central for this process and by interacting with Blinkin, link the SAC with the kinetochore, the macromolecular assembly that connects microtubules with centromeric DNA. Here, we identify the Blinkin motif critical for interaction with BUBR1, define the stoichiometry and affinity of the interaction, and present a 2.2 Å resolution crystal structure of the complex. The structure defines an unanticipated BUBR1 region responsible for the interaction and reveals a novel Blinkin motif that undergoes a disorder-to-order transition upon ligand binding. We also show that substitution of several BUBR1 residues engaged in binding Blinkin leads to defects in the SAC, thus providing the first molecular details of the recognition mechanism underlying kinetochore-SAC signaling.

## Graphical abstract

**Graphical Abstract:**

**Figure:**
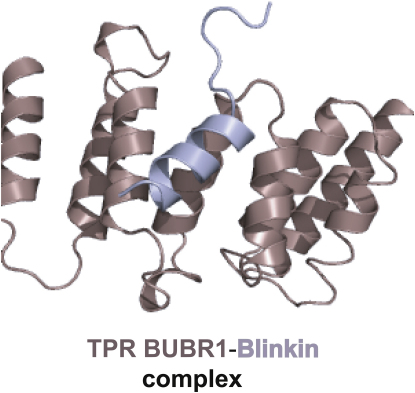

**Highlights:**

► Molecular details of the recognition mechanism underlying kinetochore-SAC signalling ► Crystal structure of a BUBR1-Blinkin complex defines an unanticipated BUBR1 region ► A novel Blinkin motif undergoes a disorder-to-order transition upon BUBR1 binding ► Mutation of BUBR1 residues that bind Blinkin lead to an impaired mitotic checkpoint

## Introduction

The spindle assembly checkpoint (SAC) is the evolutionarily conserved and essential self-monitoring system of the eukaryotic cell cycle, which prevents defects in the segregation of sister chromatids during mitosis by triggering anaphase delay in response to kinetochores incorrectly or not attached to the mitotic spindle. The multidomain protein kinase BUBR1 and its paralog BUB1 are central components of the SAC. BUBR1 forms part of the mitotic checkpoint complex (MCC) that inhibits the anaphase-promoting complex or cyclosome (APC/C)E3 ubiquitin ligase activity toward cyclin B1 and securin (Tang et al., 2001; Sudakin et al., 2001; Nilsson et al., 2008). BUBR1 also associates with unattached/incorrectly attached kinetochores and plays an important role in kinetochore microtubule interactions (Lampson and Kapoor, 2005; Elowe, 2011).

The kinetochore constitutes an essential multiprotein complex that assembles on mitotic or meiotic centromeres. This large macromolecular assembly plays a crucial role in chromosome segregation and mediates the physical contact of centromeric DNA with microtubules (Przewloka and Glover, 2009). Human Blinkin (also often referred to as KNL1, Spc105, AF15Q14, and CASC5) is a protein initially identified in *Saccharomyces cerevisiae* as a budding yeast spindle pole body component (hence the acronym Spc105) (Nekrasov et al., 2003), which in *Caenorhabditis elegans* is commonly referred to as KNL1 (kinetochore null phenotype 1) (Cheeseman et al., 2004) and as Spc105R (Spc105 related) in *Drosophila* (Przewloka et al., 2007). Blinkin is a central component of the KNL1/Mis12/Ndc80 complexes (KMN) network, the multiprotein macromolecular assembly essential for the establishment of proper kinetochore-microtubule attachments. Human Blinkin (NCBI reference code NP_653091.2) is a large protein (262 kDa) containing multiple regions of predicted low structural complexity distributed throughout the sequence. Although amino acid sequence conservation between yeast and vertebrate Blinkins is low, conserved motif repeats (S/GILK, RRVSF, and MELT) can be identified in most species (Nekrasov et al., 2003; Cheeseman et al., 2004; Przewloka et al., 2007).

Blinkin functions as a molecular scaffold to dock other proteins: its C-terminal region is the binding site of the Nsl1 and Dsn1 components of the Mis12 complex (Cheeseman et al., 2006; Kiyomitsu et al., 2007) while its N-terminal region physically interacts with the TPR domains of the SAC kinases BUB1 and BUBR1 (Kiyomitsu et al., 2007, 2011; Bolanos-Garcia et al., 2009; D'Arcy et al., 2010). The interaction of Blinkin with BUB1 and BUBR1 connects SAC signaling with the KMN network and is essential for the recruitment of both multitask kinases to the kinetochore (revised in Bolanos-Garcia and Blundell, 2011). Moreover, depletion of Blinkin of higher organisms by RNAi causes severe chromosomal segregation defects that resemble phenotypes characteristic of BUB1 and BUBR1 protein depletion (Kiyomitsu et al., 2007; Cheeseman et al., 2008).

Although recent progress has made possible determination of the hierarchy of interactions between kinetochore components, definition of the principles underlying kinetochore-mitotic checkpoint signaling requires the establishment of the structural basis of this process. To this end, we identified the region critical for interaction with BUBR1 using a yeast two-hybrid system and defined the stoichiometry and affinity of the interaction of the peptide corresponding to the binding region with BUBR1 by Nano-ESI MS and ITC. We then synthesized a chimeric peptide-BUBR1 construct and characterized the complex and chimera using 2D NMR, demonstrating that they involved equivalent interactions. We crystallized and solved the structure of human BUBR1 at 2.2 Å resolution in complex with Blinkin peptide in the form of the chimeric protein. Finally, using stable isogenic HeLa cell lines we show that specific interference with the interaction between Blinkin and BUBR1 leads to defects in the SAC and the impairment of the interaction of BUBR1 with Cdc20.

## Results

### Definition of the BUBR1 Binding Region from Blinkin

**Figure 1:**
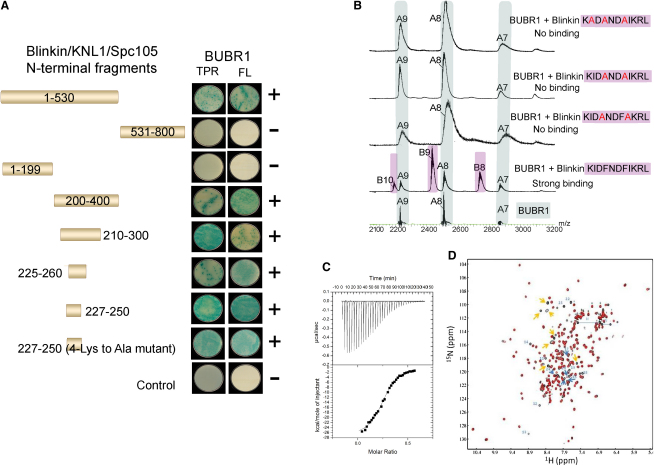
Mapping the BUBR1-Blinkin Interaction (A) BUBR1 interacts with N-terminal Blinkin fragments in a Y2H assay. Strains Y187[pGBKT7-bait] and GOLD[pGADT7-target] were mated and X-α-Gal activity assays performed on colonies grown on SD/-Ade/-His/-Leu/-Trp plates. Colony growth requires the activation of *ade* and *his* reporter genes and the blue-producing X-α-Gal reaction requires the activation of the *mel1* reporter gene to express the secreted reporter enzyme α-galactosidase. (B) Nano-ESI MS of synthetic peptides that mimic Blinkin S208-K226 confirm the interaction while peptides harbouring site-specific substitutions indicate the hydrophobic residues I213, F215, F218 and I219 are critical for binding BUBR1. (C) ITC data shows the affinity of the interaction (Kd = 9 μM); ΔH and ΔS of −2.7 kcal mol^-1^ and −64 kcal mol^-1^, respectively. Saturation of BUBR1_57-220_ with Blinkin peptide S208-K226 was achieved when BUBR1_57-220_ at concentration 27 μM was used to titrate against 200 μM of the peptide. (D) An overlay of the 2D ^1^H-^15^N HSQC spectra of BUBR1-Blinkin chimera (red) and free BUBR1 after addition of up to 2 moles of Blinkin peptide to one mol of protein. All structured amides from BUBR1 occupy an identical position in the chimera. Some extra peaks from the additional residues in the chimera are indicated: (blue, residue number shown) residues from Blinkin, which become structured upon binding and (orange) unstructured residues from the linker and extreme N terminus of Blinkin (see also Figure S1).

We first set out to define the minimal Blinkin region important for binding BUBR1 using a yeast two-hybrid (Y2H) system (see Supplemental Experimental Procedures available online). BUBR1 interactions were tested through the highly stringent Matchmaker 3 Y2H system in which positive interactions were determined by independent activation of three reporter genes (*HIS*, *ADE*, and *MEL1*) using a quadruple dropout. BUBR1_57-220_ was found to interact with fragments encompassing N-terminal Blinkin including Blinkin_1-800_, Blinkin_1-530_, Blinkin_200-400_, Blinkin_210-300_, Blinkin_225-260_, and Blinkin_227-250_ in a manner equivalent to that of full-length protein and BUBR1_57-220_ (Figure 1A), confirming that these Blinkin fragments form contacts with the TPR-containing region of BUBR1. The fact that several positively charged residues (K212, K220, R221, K223, and K226) are located in human Blinkin_208-226_ while the C-terminal region of human BUBR1_57-220_ is rich in acidic residues (E209, E211-215, and E218), suggested the possibility that the positive signal detected by Y2H reported extensive electrostatic interactions. To test this hypothesis, Y2H experiments were conducted for a Blinkin mutant where most of the basic residues (K220, R221, K223, and Lys226) were replaced by alanines. Selection under high-stringency conditions showed that this mutant binds BUBR1_57-220_ and BUBR1 full length, thus indicating that the substituted residues are not essential for complex formation (Figure 1A). A reciprocate Y2H experiment where the BUBR1 residues E211, E212, E213, and E214 were replaced by alanines rendered similar results (data not shown). Our Y2H experiments also show that fragments of N-terminal Blinkin flanking residues S208-K226, which are characterized by the presence of conserved, short motifs M(D/E)([I/L)(S/T), (S/G)ILK and RRVSF, are dispensable for the interaction. These Blinkin motifs have been shown to be unimportant for binding BUBR1 in the *Drosophila* ortholog Spc105R (Schittenhelm et al., 2009) and in humans (Kiyomitsu et al., 2011). Furthermore, very recently it has been shown that the Blinkin region encompassing residues F201 to S250 is required for binding BUBR1 and that deletion of this region impaired BUBR1 kinetochore localization (Kiyomitsu et al., 2011). The fact that these data are consistent with our highly stringent quadruple dropout Y2H data and the detailed peptide mapping, physicochemical analyses and structural information described below supports our identification of the Blinkin fragment comprising residues S208-K226 as the BUBR1 binding region.

### Biophysical Characterization Reveals a Complex of Moderate Affinity

We next investigated the contribution of conserved residues of the Blinkin region S208-K226 to the interaction with BUBR1_57-220_ using a combination of synthetic peptides that mimic Blinkin residues S208-K226 with size-exclusion chromatography, fluorescence-based thermal shift assays, Nano-ESI MS, ITC, and 2D NMR (Figures 1B–1D) (see also Supplemental Experimental Procedures and Figure S1). The micromolar binding affinity of Blinkin S208-K226 peptide for BUBR1_57-220_ (9.3 μM ± 0.4 in 1:1 stoichiometry) reflects a relatively moderate interaction. Nano-ESI MS and 2D NMR titration data confirmed the 1:1 stoichiometry of the interaction. The Tm of ligand-free BUBR1_57-220_ estimated by fluorescence-based thermal shift assays was 60°C. Addition of peptide increased the thermal stability of BUBR1_57-220_ by approximately 2°C, an observation further confirmed by far-UV CD thermal denaturation analysis (data not shown). Moreover, addition of the Blinkin S208-K226 peptide stabilized BUBR1_57-220_ to such extent that good quality HSQC spectra could be collected at 40°C for a continuous period of 10 days (Figure 1D).

Although Y2H experiments showed that substitution of Blinkin residues K220, R221, K223, and K226 by alanines did not affect BUBR1 binding, inspection of the sequence context of these residues shows they resemble a putative acetylation motif [(K/R)(K/R)xKxGK] (Kim et al., 2006). The relevance of this class of posttranslational modification for checkpoint signaling is suggested by the observation that acetylation of BUBR1 residue K250 blocks its ubiquitylation and degradation (Choi et al., 2009). However, whether acetylation of these or other residues is important for Blinkin function requires further investigation. In summary, Nano-ESI MS analyses of synthetic peptides indicate that Blinkin residues I213, F215, F218, and I219 are essential for the interaction with BUBR1_57-220_ and that substitution of Blinkin residues K220, K223 and K226 with alanines does not affect BUBR1 binding.

### A Chimera Accurately Mimics the Solution Structure of the BUBR1-Blinkin Complex

Although we had previously been able to obtain a structure of free BUBR1 (D'Arcy et al., 2010), the peptide-bound complex proved recalcitrant to crystallization trials. However, the aforementioned biochemical and biophysical characterization of the BUBR1-Blinkin interaction provided a rational basis to design a chimera construct, which comprised Blinkin residues S208-K226 linked to N terminus BUBR1 via a poly(TGS) linker. The chimera protein was expressed in *Escherichia coli* as a GST protein fusion and purified using conventional affinity chromatography conditions (see also Supplemental Experimental Procedures). The monomeric status of BUBR1_57-220_-Blinkin_208-226_ was consistent with line widths in 1D ^1^H NMR spectra and analytical size exclusion chromatography in which BUBR1_57-220_-Blinkin_208-226_ eluted as a single species at a volume corresponding to 1.4 times the mass predicted for a compact, globular monomer (data not shown). Backbone assignment and side-chain resonances of ^15^N/^13^C N-terminal BUBR1 in the unbound state and in complex with an unlabelled Blinkin mimic peptide will be published elsewhere (P.J.S., E.C., and V.M.B-G., unpublished data).

In order to establish whether the chimeric construct represents the true conformation of the complex in solution, ^15^N, ^13^C-labeled native BUBR1 and BUBR1-Blinkin chimera were expressed for more in-depth NMR analysis. Backbone atoms of native BUBR1 were assigned using conventional methods and the protein titrated with Blinkin S208-K226 peptide. ^1^H-^15^N HSQC spectra upon addition of up to 2 moles of peptide to one mole of protein revealed extensive shift changes in BUBR1, the majority of which were in slow-exchange on the NMR timescale in line with the affinity measured by ITC (Figure 1D). Importantly, comparison of the HSQC spectrum of the bound state with that of the chimera revealed that all structured BUBR1 signals overlay with those of the native BUBR1-Blinkin complex (Figure 1D). A number of additional peaks are observed in the chimera, which were shown to arise from the extra linker and Blinkin residues S208-K226. As expected, nonrandom coil shifts are observed for residues corresponding to the newly formed Blinkin helix as judged by the chemical shift index (CSI) (see also Figure S1A). Furthermore, the additional linker is characteristic of an unstructured region, with random-coil chemical shifts and reduced ^1^H-^15^N heteronuclear NOE values indicative of elevated picosecond-timescale motion (Figure S1B) relative to the majority of BUBR1 and the Blinkin helix. As the NMR frequency is exquisitely sensitive to chemical environment and local conformation, the observation that the chimera spectrum is essentially identical to that of the native BUBR1-Blinkin complex, together with the CSI and dynamics data, indicate that the chimera construct mimics the native BUBR1-Blinkin interaction.

### The Crystal Structure Reveals an Unexpected Binding Mode

**Figure 2:**
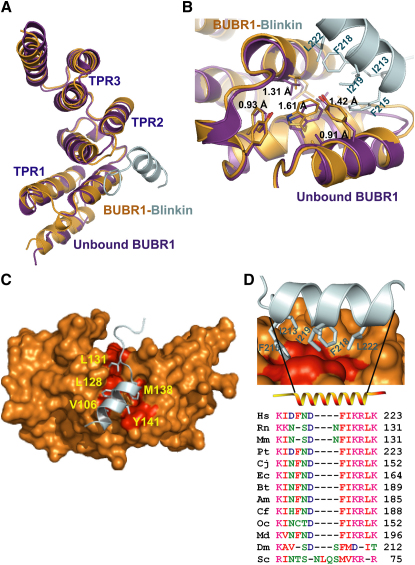
The 2.2 Å Crystal Structure of the BUBR1-Blinkin Complex Reveals an Unexpected Mode of Binding (A) Superposition of the free (magenta) and Blinkin-bound TPR BUBR1 crystal structures (orange) reveals that little conformational changes occur upon ligand binding. A closeup of TPR2 BUBR1, where the slight conformational changes that result from Blinkin binding are most noticeable. (B) Surface representation of TPR BUBR1; the hydrophobic residues relevant for the interaction of this protein with Blinkin (blue ribbon) are highlighted in red. (C) Blinkin residues I213, F215, F218 and I219 bind a shallow hydrophobic groove. (D) Amino acid sequence alignment of the Blinkin BUBR1 binding region reveals a high conservation of these residues in higher organisms. Each molecule representation was generated with Pymol (DeLano, 2002). The aligned sequences are from *Homo sapiens* (Hs); *Rattus norvegicus* (Rn); *Mus musculus* (Mm); *Pan troglodytes* (Pt); *Callithrix jacchus* (Cj); *Equus caballus* (Ec); *Bos taurus* (Bt); *Ailuropoda melanoleuca* (Am); *Canis familiaris* (Cf); *Oryctolagus cuniculus* (Oc) and *Monodelphis domestica* (Md); *Drosophila melanogaster* (Dm); and *Saccharomyces cerevisiae* (Sc).

**Figure 3:**
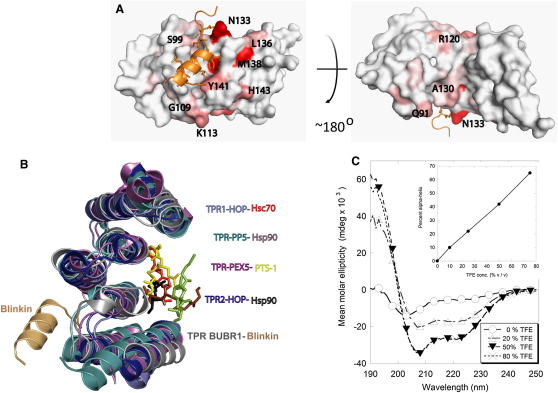
Blinkin Shows a Novel BUBR1 Binding Motif (A) NMR chemical shift changes are consistent with the location of the peptide in the chimera. BUBR1-Blinkin chimera is shown as a surface representation with chemical shift changes upon binding of Blinkin peptide to native BUBR1 indicated as a white-red ramp: white, no change/data not available; red, largest shift change upon peptide binding. The largest changes in chemical environment are on the convex face and are consistent with the location of the peptide in the crystal, although some shifts propagate through to the concave face (see also Figure S1). (B) Blinkin binds a region that differs from that observed in TPR-peptide complexes of high structure similarity. (C) Far-UV circular dichroism spectra reveal a predominantly disorder structure of Blinkin peptide S208-K226 in aqueous solutions. Gradual addition of TFE resulted in a dramatic conformational change and the formation of a predominantly α-helical structure.

Single crystals of Blinkin_208-226_-BUBR1_57-220_ were obtained in two different conditions by conventional vapor diffusion hanging drop methods. Crystals from one condition were of space group *C2* and diffracted to 2.2 Å. The structure of Blinkin_208-226_-BUBR1_57-220_ was solved by molecular replacement using the TPR-containing domain of human BUBR1 (PDB 2WVI) (D'Arcy et al., 2010) with a final *R* factor of 19% and *R*free of 24% (Table 1) (see also Supplemental Experimental Procedures). A comparison of the crystal structure of BUBR1 in the ligand free form (D'Arcy et al., 2010) and in the complex shows that BUBR1 undergoes little conformational change upon binding Blinkin and preserves the features characteristic of TPR motifs, including a concave inner surface and a subtle right-handed super-helical twist of the entire structure (Figure 2A). In the complex, the Cα atoms of the BUBR1 residues V106, W125, Y139, Y141, and L142 show displacements of 1.31, 1.61, 1.02, 1.42, and 0.91 Å, respectively, with respect to the unliganded protein (Figure 2B). A shallow cavity lined by BUBR1 residues L102, V106, Y116, F121, W125, L128, L131, Y139, Y141, and L142 (Figure 2C) suggests that these residues contribute significantly to the interaction with Blinkin. Electron density of the Blinkin residues N211-G225 was clearly visible and unequivocally shows that the side chains of Blinkin residues I213, F215, F218, and I219 form part of a short α helix defined by residues F215-T224 (Figure 2D) that runs parallel to the long axis of BUBR1 TPR1 and TPR2. NMR data show that the minor conformational changes of BUBR1 residues located relatively distant from the protein interface observed in the crystal structure also occur in solution (Figure 3A). It is also clear that the bound form of TPR BUBR1 maintains its unique features including a loop insertion in the first TPR unit that defines a shallow groove, the insertion of a 3_10_ helix between the second and third TPR tandem repeats, and the noncanonical packing interactions established between the α helices of the second TPR unit (D'Arcy et al., 2010).

TPR BUBR1 shows the closest structural similarity to TPR BUB1 (PDB 2WVI and 3ESL, respectively) and high structural similarity with the TPR domains of protein phosphatase 5 (PP5), Hsp90 organizing protein (HOP) and PEX, despite little amino acid conservation in equivalent positions. Therefore, the similar topology suggests that BUBR1 and Blinkin may interact in a mode similar to that observed in complexes formed between TPR PP5 and a Hsp90 peptide (PDB 2BUG) (Cliff et al., 2006); TPR HOP-Hsc70 peptide (PDB 1ELW) (Scheufler et al., 2000); TPR HOP-Hsp90 peptide (PDB 1ELR) (Scheufler et al., 2000); and TPR PEX5 in complex with a peroxisomal targeting signal-1 (PTS1) peptide (PDB 1FCH) (Gatto et al., 2000). In these examples, a central region of the concave face defined by the TPR units forms a “cradle” where the peptide binds (revised in Bolanos-Garcia and Blundell, 2011). Unexpectedly, the BUBR1-Blinkin complex structure reveals a mode of interaction that is very different from that observed in the structurally similar TPR-peptide complexes (Figure 3B).

Several positions defining the shallow hydrophobic cavity of BUBR1 have conserved residue types in BUB1. For example, the BUBR1 residues V106, L128, Y139, Y141, and L142 are substituted by hydrophobic residues in BUB1 (M53, F75, F86, F88, and L89, respectively). However, other residues vary greatly. For instance, BUBR1 residues E107, Y116, and W125 are substituted by K54, H63, and C72 in human BUB1, respectively (Figure S2). Such amino acid sequence variation suggests that BUB1 recognizes Blinkin residues different from those engaged in binding BUBR1 (Kiyomitsu et al., 2011).

Polar contacts contribute to stabilize the BUBR1_57-220_-Blinkin_208-226_ complex: salt bridges are formed between Blinkin residue R221 and BUBR1 residues E103 and E107 while hydrogen bonds are established between BUBR1 L131, a residue that is located in α helix A of TPR2, with Blinkin residue I213. Residue N133, which is located in a loop connecting TPR2 α helices A and B, establishes a hydrogen bond with the main chain Blinkin residue I213. Interestingly, several BUBR1 residues important for binding Blinkin (i.e., residues E107, L128, L131, Y141, and L142) are conserved in human and murine BUBR1 but not in yeast or worms, suggesting that sequence variations in the BUBR1 protein interface accounts for species specific interactions between BUBR1 and Blinkin orthologs. Similarly, Blinkin residues within the BUBR1 binding region are highly conserved in a large number of higher organisms but show more variation in flies and yeast (Figure 2D). Blinkin_208-226_ is disordered prior to binding as suggested by secondary structure prediction algorithms (data not shown) and demonstrated by circular dichroism experiments (Figure 3C) (see also Supplemental Experimental Procedures). However, the structure reveals that this Blinkin region undergoes a dramatic disorder-to-order transition upon BUBR1 binding, a feature also observed in TFE-titration experiments of Blinkin mimic peptides monitored by circular dichroism (Figure 3C and inset).

In an attempt to relate structure with the binding affinity of the interaction, we calculated the interfacial surface area of the complex with PDBePISA. This interactive tool calculated a total surface area of 9140 Å^2^ and 2100 Å^2^ for individual BUBR1_57-220_ and Blinkin_208-226_, respectively, and an interface area of 1150 Å^2^ for the complex. In the context of a recent classification (Kastritis et al., 2011) of protein-protein complexes with Kd ranging between 10^−5^ and 10^−14^ M and our ITC data, we conclude that the Blinkin-BUBR1 interaction is one of medium affinity. Such affinity correlates well not only with the Kd determined for other complexes of a similar interface area that undergo minor conformational changes upon ligand binding (Kastritis et al., 2011) but also with the transient nature of the SAC response in cells with an unsatisfied mitotic checkpoint.

### The interaction between BUBR1 and Blinkin Is Required for a Functional SAC

Previous studies have demonstrated that Blinkin is required for a functional mitotic checkpoint potentially through the recruitment of BUB1 and BUBR1 to the kinetochore (Kiyomitsu et al., 2007, 2011). The studies, which were largely based on truncated constructs for both in vivo localization experiments and the yeast two-hybrid system, did not discriminate between a requirement of BUB1-Blinkin interaction and that of BUBR1-Blinkin. The structure of Blinkin-BUBR1 complex allows a closer inspection of the role of specific BUBR1 residues for the interaction and the impact of residues substitution in SAC signaling.

To this effect we created stable isogenic HeLa cells lines expressing siRNA resistant Venus-BUBR1 WT, Venus BUBR1 L128A/L131A and Venus BUBR1 Y141A/L142A. Analysis of the structure of the Blinkin-BUBR1 complex suggests that the two double mutants are likely to disrupt the interaction with Blinkin. As a control we included the KEN box of BUBR1 where all three residues were mutated to alanine (Venus BUBR1 KEN26AAA) known to have a defective checkpoint (Sczaniecka et al., 2008; Malureanu et al., 2009).

**Figure 4:**
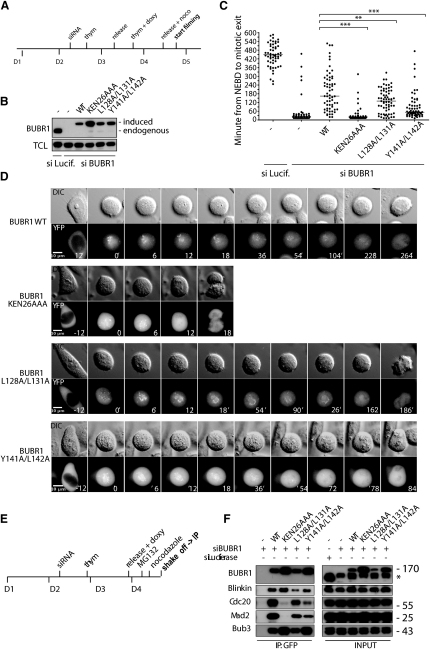
The Interaction between BUBR1 and Blinkin Is Required for a Functional SAC (A) Synchronization protocol for RNAi depletion of BUBR1 and rescue with mutants. Time is shown in days (D). (B) Western blot for BUBR1 showing depletion of endogenous BUBR1 and level of expression of the different mutants (see also Figure S3). (C) Plot showing time spent in mitosis in the presence of 100 nM nocodazole with each dot representing a single cell analyzed by time-lapse microscopy and bar indicating the median. Time is scored as the time from nuclear envelope breakdown (NEBD) to time of mitotic exit and for each condition 60 cells have been analyzed. The statistical significance is indicated above as determined by the Mann-Whitney test. The time spent in mitosis in the Luciferase control is underestimated as 49 out of 60 cells did not exit mitosis during filming. (D) Representative still images from the different cell lines analyzed. Time is in minutes and t = 0 is at NEBD. (E) Synchronization protocol used for the immunodepletion experiment. (F) Immunopurified BUBR1 complexes analyzed for the presence of Blinkin, Cdc20, Mad2, and Bub3. In the input the asterisks indicates an unspecific band from previous probing of the blot.

The cell lines were synchronized using a double thymidine arrest protocol. Endogenous BUBR1 was depleted for 48 hr and then exogenously induced for 24 hr before filming the cells using time-lapse microscopy in the presence of 100 nM nocodazole (Figures 4A–4D; Movies S1–S4). Control cells were arrested under these conditions while BUBR1 RNAi-treated cells failed to arrest. Importantly, BUBR1 arrest could be restored by expressing Venus-BUBR1 WT but not Venus-BUBR1 KEN26AAA, thus demonstrating the effectiveness of the complementation assay (Figure 4D; Movies S1 and S2). The arrest induced by complementation with Venus-BUBR1 WT is not as strong as that of control-treated cells indicating that full rescue could not been achieved. This feature may be the result of a lower expression level of exogenous BUBR1, interference caused by the protein tag or both. However, the clear difference between Venus-BUBR1 WT and Venus-BUBR1 KEN26AAA allowed us to address the role of the BUBR1-Blinkin interaction in the SAC.

Both BUBR1 double mutants L128A/L131A and BUBR1 Y141A/L142A showed the expected cytoplasmic localization before cells entered into prometaphase and kinetochore accumulation in prometaphase. Importantly, both L128A/L131A and Y141A/L142A point mutants failed to mount a SAC arrest to the same extent as BUBR1 WT, thus suggesting these residues play an important role in the BUBR1-Blinkin interaction (Figure 4D; Movies S3 and S4), a notion that is supported by analytical gel filtration chromatography coupled to mass spectrometry, NMR data, and two-hybrid assays (Figures 3B–3D). To rule out the possibility that a defective SAC was an artifact resulting from protein misfolding, both L128A/L131A and Y141A/L142A double mutants were overexpressed in *E. coli* and characterized by analytical gel filtration, far-UV CD and NMR. The analyses confirmed that the two mutants exhibit a native, monomeric state and secondary structure and thermal stability that are similar to BUBR1 WT (Figure S3A). As expected, the BUBR1 double mutants L128A/L131A and Y141A/L142A have a lower affinity for binding the Blinkin peptide compared to the WT as revealed by analytical gel filtration (data not shown) and nano ESI MS (Figure S3B).

In particular, BUBR1 Y141A/L142A displayed an almost complete lack of checkpoint, a feature that was also observed when cells were not challenged with nocodazole (data not shown). We set out to investigate whether the impaired SAC function of these BUBR1 mutants was related to the ability to bind Cdc20. For this, we immunopurified each BUBR1 double mutant in the absence of endogenous BUBR1 using GFP-Trap according to the strategy outlined in Figure 4E. As expected, BUBR1 WT showed binding to Cdc20, Mad2 and Blinkin while BUBR1 KEN26AAA completely lost its interaction with Cdc20 and Mad2 (Figure 4F). Importantly BUBR1 L128A/L131A and BUBR1 Y141A/L142A had a diminished binding to Cdc20, Mad2 and Blinkin. The impaired binding to these proteins is more evident in the case of the BUBR1 Y141A/L142A mutant, an observation that is in good agreement with our live cell analyses (Figure 4D). These experiments reveal that disrupting the interaction between BUBR1 and Blinkin, which results in a failure in the SAC, also affects the binding of BUBR1 to Cdc20.

## Discussion

We have investigated the interaction of Blinkin with BUBR1 using a variety of methods: the Y2H system, peptide binding mapping studies, CD and ITC measurements, nano ESI MS, NMR, X-ray crystallography, and time-lapse microscopy of BUBR1 mutants expressed stably in HeLa cells. Our multidisciplinary approach allowed us to identify Blinkin I213-L222 as the minimal residues required for binding BUBR1 and to provide binding affinity data. Moreover, when this manuscript was in preparation, a Blinkin fragment (residues F201-S250) was reported as sufficient for the interaction with BUBR1 and its deletion was shown to compromise SAC function (Kiyomitsu et al., 2011). The comparatively larger Blinkin region thus identified is in good agreement with our Y2H experiments, in which reciprocal interactions were tested under highly stringent conditions, and our detailed biochemical, biophysical, structural, and functional analyses of the interaction. However, our structural studies clarify some recent findings as we show that Blinkin residues I213, F215, F218, I219, R221, and L222 are essential for the interaction with human BUBR1 and help to define precisely a novel BUBR1 binding motif (**I-**x-**F-**x-x-**F-I-**x-**R**-**L**) as opposed to the KI motif (KI[D/N]xxxF[L/I]xxLK, residues 212 to 223) inferred from Y2H studies of truncated constructs (Kiyomitsu et al., 2011).

The structure indeed reveals that the interaction of Blinkin_208-226_ with BUBR1_57-220_ is derived from complementary hydrophobic interfaces implicating Blinkin residues I213, F215, F218, I219, and L222. Blinkin residue R221 contributes to the interaction through the establishment of salt bridges with BUBR1 residues E103 and E107 while other Blinkin residues that show good conservation in higher organisms including those that form part of the KI motif K212, D214, N216, and D217, are not engaged in binding BUBR1. Previous Y2H experiments indicated that single substitution of BUBR1 residues P119, R130 and S157 as well as those defining the GIG motif (i.e., G146 and G148) by alanine does not have an effect on the interaction with Blinkin and that a similar substitution of residues L126, E161, and R165 weakened the interaction (D'Arcy et al., 2010). The structure shows that BUBR1 residue L126 is located in close proximity to residues W125 and L128, which contribute to define the shallow groove whereas the GIG motif is mapped onto the loop region linking TPR2 and TPR3. Thus, substitution of residue L126 by alanine and double and triple substitutions of residues defining the GIG motif are expected to disrupt the architecture of the ligand-binding site. Residues E161 and R165 are located approximately 11 Å away from the shallow groove described above, suggesting that substitution of these residues with alanine most likely impaired Blinkin binding by destabilizing interactions between TPR units. This idea is supported by the fact that two residues located in close vicinity, W158 and Y162, are engaged in stacking interactions with others located at TPR consensus positions (D'Arcy et al., 2010).

The structure allows the mapping of residue substitutions that have been associated with cancer. For instance, the substitution E166D initially identified in patients with adult T cell leukemia/lymphoma (Ohshima et al., 2000), and Y155C, a substitution associated with mosaic-variegated aneuploidy (Suijkerbuijk et al., 2010), both are mapped onto the third TPR unit. The distant position of these residues relative to the protein-peptide interface suggests that a similar destabilization of helix-helix contacts may account for the deleterious outcome of these residue substitutions.

Remarkably, the BUBR1-Blinkin structure revealed an unanticipated protein-protein interface in which Blinkin interacts with a shallow hydrophobic groove located in the convex side of the BUBR1 helical bundle (Figure 3B). This structural feature suggests that TPR BUBR1 might contain more than one protein-binding site, a likely possibility considering the various roles of this kinase in SAC signaling (Bolanos-Garcia and Blundell, 2011; Elowe, 2011). In any case, the novel interface defined by the Blinkin-BUBR1 complex expands the repertoire of protein-protein interaction sites recognizable in a TPR motif. Moreover, the unique mode of interaction of TPR BUBR1 with Blinkin, the high divergence of its amino acid sequence from a canonical TPR motif and the unique structural features of TPR BUBR1 (and TPR BUB1) suggest that these SAC kinases represent a distinct structural class of TPR-containing proteins. Moreover, studies conducted in stable cell lines show that mutation of BUBR1 residues defining the shallow hydrophobic grove impaired the SAC and the incorporation of BUBR1 into checkpoint complexes (Figures 4A–4F) further indicating that the interaction between BUBR1 and Blinkin is important for a functional SAC. Considering that BUBR1 L128A/L131A and BUBR1 Y141A/L142A still localize to the kinetochore and that these mutants bind the Blinkin mimic peptide with much lower affinity than BUBR1 WT, it seems likely that multiple sites of contact must be established between BUBR1 and Blinkin in order to make the interaction productive and/or that additional contacts between BUBR1 and the kinetochore may exists. For instance, we have evidence suggesting that BUB3 can also contribute to the interaction with Blinkin (unpublished data). We favor a model in which BUBR1 binds Blinkin and potentially gets posttranslationally modified by kinetochore localized mitotic kinases allowing its incorporation into complexes with Cdc20. However, it cannot be ruled out that Cdc20 and Blinkin share an overlapping binding site on BUBR1 and that this is the cause of the checkpoint defect we observed in the aforementioned BUBR1 double mutants. Further experiments should aim to define more precisely the causality of Blinkin and checkpoint protein binding to BUBR1 mutants in regards to mitotic checkpoint deficiency.

Our mapping studies show that Blinkin_208-226_ does not require a conformation dictated by tertiary, intramolecular interactions to bind BUBR1. Instead, it appears that this motif undergoes an important folding transition upon binding, analogous to that described by Dyson and Wright (2002). We show that a hydrophobic environment, achieved experimentally by addition of 2,2,2-trifluoroethanol to the aqueous buffer solution, promotes α helix formation of this peptide (Figure 3C), which suggests that in vivo, binding of this fragment is coupled with α helix formation and the recognition of a specific hydrophobic surface in BUBR1_57-220_. Furthermore, it is likely that upon BUB1 and BUBR1 binding N-terminal Blinkin undergoes conformational changes that may affect its interaction with other ligands. In any case, the biophysical evidence derived from binding mapping studies of Blinkin-mimic synthetic peptides bearing site-specific substitutions together with the characterization in vivo of BUBR1 site-specific mutants and atomic details of the mode of interaction prompt us to suggest that complex formation underlie a sequential zipper or Velcro mechanism in which Blinkin residues I213, F215, F218, and I219 dock into the BUBR1 pockets before the residue R221 establishes a salt bridge with BUBR1 E107. An important implication of such a sequential zipper or Velcro mechanism is that cooperative interactions may lead to increased specificity and more sensitive regulation and might occur in a similar mode to that observed in other signaling systems, such as ligase IV-Xrcc4 and BRCA2-Rad51 complexes (Blundell et al., 2002; Ochi et al., 2010; Bolanos-Garcia et al., 2011). Although fly and yeast Blinkin orthologs show a greater amino acid sequence divergence than higher organisms, it is expected that features relevant for the interaction with SAC kinases are conserved. Indeed, the fly Blinkin ortholog Spc105R, which seems to have diverged faster than other invertebrates (Schittenhelm et al., 2009), contains a putative BUBR1 binding motif that is defined by the residues K202 to L214 (Figure 2D).

**Figure 5:**
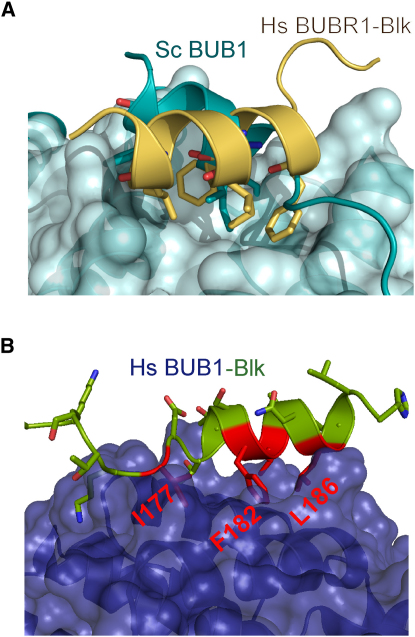
BUB1 and Blinkin/KNL1/Spc105 Complexes Seem to Show a Similar Mode of Interaction (A) Superposition of the crystal structures of yeast BUB1 and BUBR1-Blinkin showing that the intermolecular interactions observed in the former molecule occur in a similar fashion as those defining the BUBR1-Blinkin complex. (B) Structure model of human TPR BUB1 (blue) in complex with a putative Blinkin/KNL1 BUB1 binding motif residues E172-H189, predicts that hydrophobic residues I177, F182, L183, and L186 (shown in stick representation) and possibly T179 (not shown) play an important role in the interaction.

The crystal structure of TPR BUB1 from budding yeast reveals that the flexible C terminus of this domain (including residues F222, I223, F226, and L227 in helix 10) folds back to form a hydrophobic core with residues in helices 3, 4 and 5 (I93, W111, Y114, and Y132) (Bolanos-Garcia et al., 2009). Interestingly, this is remarkably similar to the hydrophobic cluster we observe in the BUBR1-Blinkin complex (Figure 5A). Moreover, the high structure conservation between TPR BUB1 and TPR BUBR1 suggests that conserved hydrophobic residues in the Blinkin region reported recently to bind human BUB1 (i.e., residues M151-N200) (Kiyomitsu et al., 2011) may define a similar binding motif (I_177_xT_179_xxF_182_L_183_xxL_186_) and mode of interaction as that of BUBR1-Blinkin (Figure 4B). If true, it will be important to establish how subtle variations of the residues lining the shallow hydrophobic grooves of BUB1 and BUBR1 contribute to dictate specificity to the interaction.

**Figure 6:**
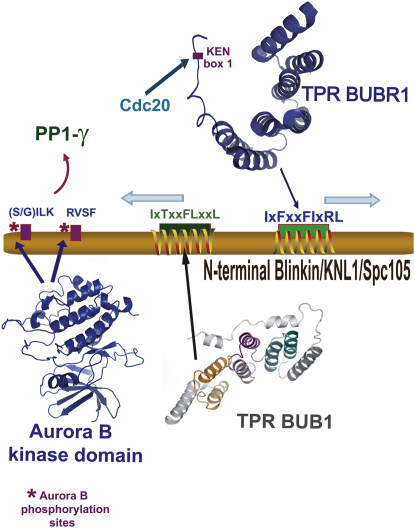
Functional Implications of the BUB1/BUBR1-Blinkin Interaction Binding to the kinetochore stimulates BUBR1 kinase activity, which results in prolonged mitotic arrest. Blinkin N-terminal residues defining the I177xTxxFLxxL186 motif are predicted to constitute a putative BUB1 binding site. The conserved Blinkin motif I213xFxxFIxRL222 is responsible of binding BUBR1 in a process that involves important disorder to order transitions. Thus, local conformational changes upon BUB1 and/or BUBR1 binding (blue arrows) are likely to affect other interactions involving this Blinkin region. For instance, N-terminal Blinkin contains a conserved motif that binds protein phosphatase 1 γ (PP1 γ), an interaction that is required for the targeting of PP1 to the outer kinetochore. The Blinkin-PP1 γ interaction is disrupted by Aurora B-dependent phosphorylation of Blinkin, which targets residues of two conserved motifs (S/GILK) and (RVSF). The diversity of key protein-protein interactions mediated by Blinkin evidences the complexity of the molecular mechanisms mediating kinetochore-mitotic checkpoint signaling.

Although the crystal structure of the BUBR1-Blinkin complex shows that the distal, extended N-terminal and C-terminal residues of the S208-K226 Blinkin fragment are not required to interact with the bound BUBR1_57-220_ domain, the length of this linker and the need to nucleate folding during binding would allow unbound flexible regions to be recognized by specific kinases and/or phosphatases (Figure 6). The observation that graded levels of microtubule-binding activity arise from different phosphorylation states of components of the KMN network, including Blinkin (Welburn et al., 2010), seems to support the notion that not only specificity but also transience can be achieved by this mechanism.

In summary, the organization of the Blinkin-BUBR1 complex provides structural details of the communication between the SAC and the kinetochore and reveals important features of how molecular recognition is achieved in this signaling system. Undoubtedly, further molecular insights into how the enrichment of BUB1 and BUBR1 in the kinetochore affects the interaction of this multisubstrate platform with Aurora B, PP1 γ, and microtubules will provide important clues about the regulation of kinetochore-mitotic checkpoint signaling and the principles that govern kinetochore assembly and disassembly in a spatial-temporal framework.

## Experimental Procedures

Details of the methods and procedures used in this work can be found in the Supplemental Experimental Procedures and include descriptions of protein expression, purification, and crystallization; Y2H analysis; mass spectrometry; circular dichroism; peptide synthesis; X-ray diffraction data collection and structure solution; NMR experiments; generation of stable HeLa cell lines; RNAi experiments; and time-lapse microscopy.

## Figures and Tables

**Table 1:** Crystallographic Data Collection and Refinement Statistics

X-Ray Diffraction Data	
Space group	C2
Unit cell: *a, b, c* (Å), beta (°)	124.40, 40.14, 75.39, 91.38
Resolution range (Å)	38.20–2.20 (2.30–2.20)
*R*_sym_^a^ (%)	11.9 (46.5)
Completeness (%)	99.9 (100)
Number of unique reflections	19,266 (2379)
Average redundancy	8.8 (6.3)
Average intensity, < I/s(I) >	14.1 (2.9)
Wilson *B*-factor (Å^2^)	26.1

**Refinement**	

Resolution range (Å)	38.20–2.20
Number of reflections: work/test	18,278/985
*R*_cryst_^b^ (%)	19.3
*R*_free_^c^ (%)	24.7
Number of nonhydrogen atoms Protein Water	3046 2796 250

**Model Quality**	

*Estimated coordinate error*^d^*(Å)*	0.225
*Rmsd bonds (Å)*	0.011
*Rmsd angles (°)*	1.116
Ramachandran plot analysis^e^ (number of residues in)	
Preferred regions	311
Allowed regions	4
Disallowed regions	3

Values in parentheses show the corresponding statistics in the highest resolution shell.

a *R*_sym_ = Σ_h_|I _h_ − < I > |/Σ _h_I _h_, where I_h_ is the intensity of reflection h, and < I > is the mean intensity of all symmetry-related reflections.

b *R*_cryst_ = Σ‖*F*_obs_|-|*F*_calc_‖/Σ|*F*_obs_|, *F*_obs_ and *F*_calc_ are observed and calculated structure factor amplitudes.

c *R*_free_ as for *R*_cryst_ using a random subset of the data (about 5%) excluded from the refinement.

d Estimated coordinate error based on the *R*_free_ value as calculated by REFMAC (CCP4, 1994).

e Calculated with PROCHECK (Laskowski et al., 1993).

## Accession Numbers

The coordinates of the BUBR1-Blinkin complex, together with structure factors, have been deposited in the Protein Data Bank under accession number 3SI5. The full set of chemical shifts of BUBR1 upon titration with a Blinkin mimic peptide has been deposited in the BioMagResBank database (http://www.bmrb.wisc.edu), accession number 17960.

## Supplemental Information

Document S1. Three Figures and Supplemental Experimental Procedures

Movie S1. Data of cells expressing native BUBR1 protein

Movie S2. Data of cell expressing the BUBR1 mutant (KEN26AAA)

Movie S3. Data of cell expressing the BUBR1 double mutant (L128A/L131A)

Movie S4. Data of cell expressing the BUBR1 double mutant (Y141A/L142A)
