# Structure of a Blinkin-BUBR1 Complex Reveals an Interaction Crucial for Kinetochore-Mitotic Checkpoint Regulation via an Unanticipated Binding Site

**DOI:** 10.1016/j.str.2011.11.011

**Published:** 2011-12-07

**Authors:** Victor M. Bolanos-Garcia, Tiziana Lischetti, Dijana Matak-Vinković, Ernesto Cota, Pete J. Simpson, Dimitri Y. Chirgadze, David R. Spring, Carol V. Robinson, Jakob Nilsson, Tom L. Blundell

**Affiliations:** 1 Department of Biochemistry, University of Cambridge, Cambridge CB2 1GA, UK; 2 The NNF Center for Protein Research, Faculty of Health Sciences, University of Copenhagen, Blegdamsvej 3B, DK-2200 Copenhagen, Denmark; 3 Department of Chemistry, University of Cambridge, Cambridge CB2 1EW, UK; 4 Department of Life Sciences, Imperial College London, London SW7 2AZ, UK; 5 Department of Chemistry, University of Oxford, Oxford OX1 3QZ, UK

## Main Text

(Structure *19*, 1691–1700; November 9, 2011)

The original article unfortunately included two errors: one in the affiliations and one figure correction. The affiliation for David R. Spring was listed incorrectly, but appears correctly, above. Panel B in Figure 3 was printed without the legend “Cdc16h-Cdc26 and the ribbon diagram representing the protein Cdc16h.” The revised panel is below.

## Figures and Tables

**Figure 3B:**
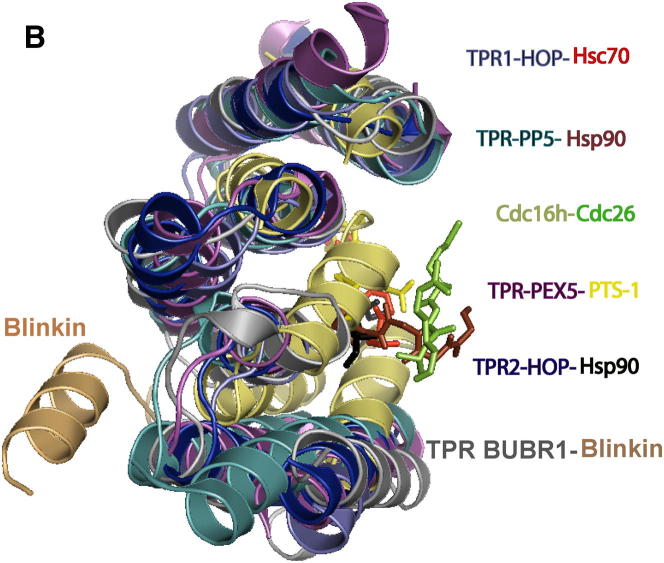
Blinkin Shows a Novel BUBR1 Binding Motif

