# Supplementary material for: Structure of a Blinkin-BUBR1 Complex Reveals an Interaction Crucial for Kinetochore-Mitotic Checkpoint Regulation via an Unanticipated Binding Site
Source: Structure. 2011 Nov 9;19(11-2):1691–700. doi: 10.1016/j.str.2011.09.017 (PMC3267040; doi:10.1016/j.str.2011.09.017)
Supplement: Document S1. Three Figures and Supplemental Experimental Procedures [file mmc1.pdf]

Structure, *19*

Supplemental Information

Structure of a Blinkin-BUBR1 Complex Reveals an Interaction Crucial for Kinetochore-Mitotic Checkpoint Regulation via an Unanticipated Binding Site

Victor M. Bolanos-Garcia, Tiziana Lischetti, Dijana Matak-Vinković, Ernesto Cota, Pete J. Simpson, Dimitri Y. Chirgadze, David R. Spring, Carol V. Robinson, Jakob Nilsson, and Tom L. Blundell

<u>Inventory of Supplemental Information</u>

Supplemental Figures

Suppl. Figure 1. Shows details of NMR data depicted in Figure 1, panel D.

Suppl. Figure 2. Shows the amino acid sequence similarities between BUB1 and BUBR1 (known as Mad3 in yeasts).

Suppl. Figure 3. Present additional data of the characterization of the BUBR1 mutants (L128A-L131A and Y141A-L142A)

Supplementary Movies 1-4. The movies show time-lapse microscopy of BUBR1 mutants.

Movie 1. Data of cells expressing native BUBR1 protein.

Movie 2. Data of cell expressing the BUBR1 mutant (KEN26AAA).

Movie 3. Data of cell expressing the BUBR1 double mutant (L128A/L131A).

Movies 4. Data of cell expressing the BUBR1 double mutant (Y141A/L142A).

Supplemental Data

Supplemental Figures

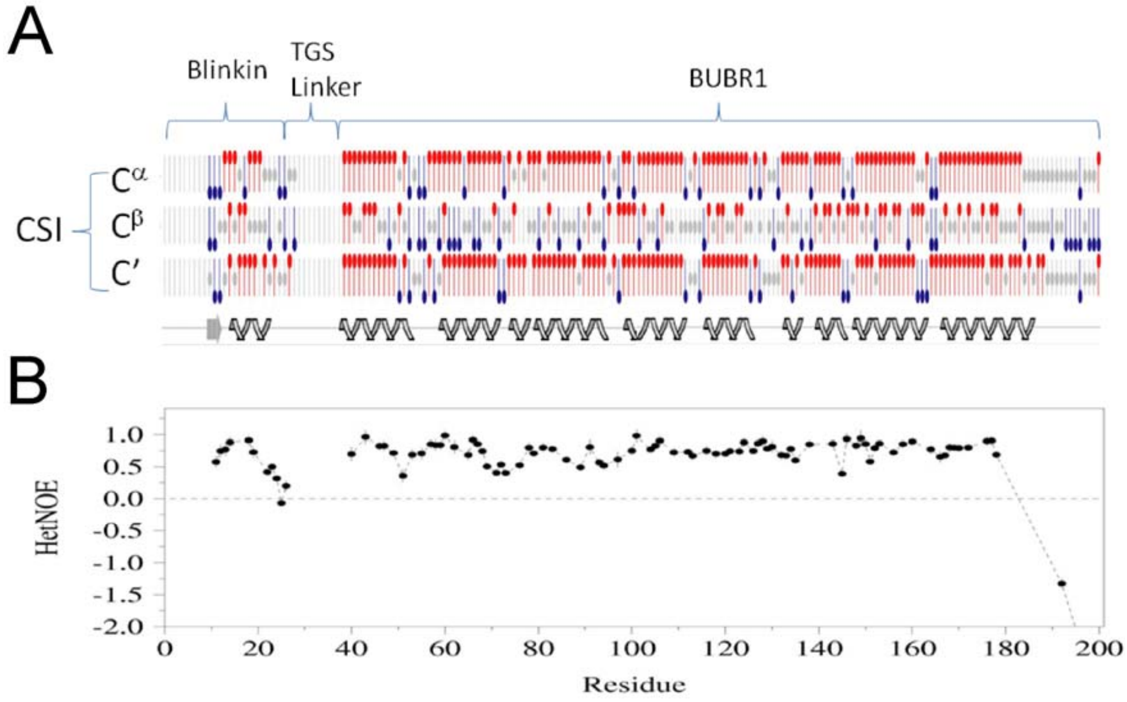

Suppl. Figure S1, related to Figure 1. NMR dynamics and chemical shift data confirm that Blinkin adopts a rigid helical structure in solution. (Top) Chemical shift index (CSI) for the BUBR1-Blinkin complex. Red lollipops indicate helical deviations from random coil, blue indicate strand-like deviations. The consensus secondary structure is shown below. Shifts for BUBR1 are taken from the native bound state and for Blinkin from the chimera. (Bottom) ^{1}H-^{15}N heteronuclear NOE. The majority of BUBR1 and the helical region of Blinkin have similar NOE values of 0.7-0.9 indicative of a rigid tumbling molecule. Residues towards the C-terminal end of Blinkin and the start of the TGS linker have decreased NOEs indicative of conformational disorder on the picoseconds timescale. The majority of the TGS linker could not be analysed due to signal overlap with other residues within the linker, as expected for an unstructured region.

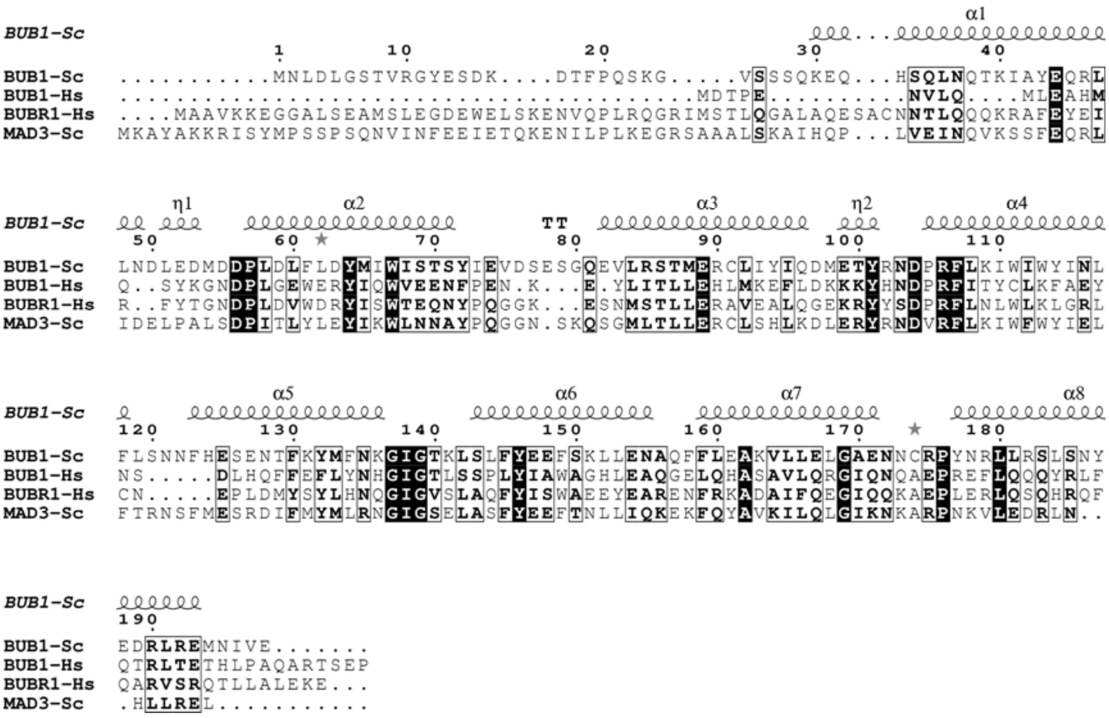

Suppl. Figure S2, related to Figure 3. Amino acid sequence alignments of BUB1 and BUBR1(Mad3) from *Saccharomyces cerevisiae* (Sc) and *Homo sapiens* (Hs). The secondary structure elements show above the aligned sequences are from the crystal structure of TPR BUB1 (PDB 3ESL). Figure generated with the programme ESPript (Gouet et al., 1999).

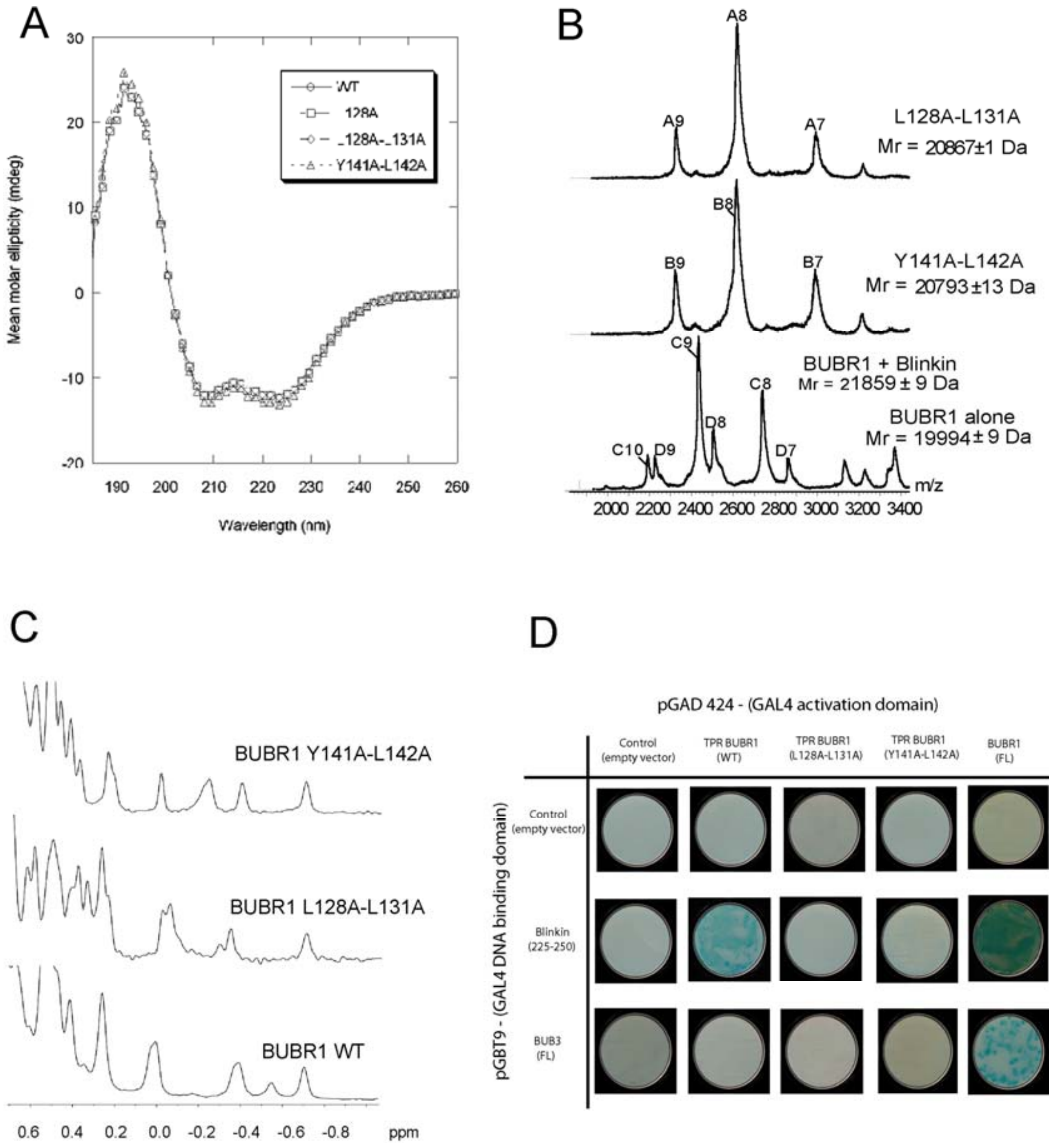

Suppl. Figure S3, related to Figure 4. Characterization of BUBR1 mutants. (**A**) The single (L128A) and two double mutants (L128A-L131A and Y141A-L142A) were studied by far-UV CD after purification by gel filtration chromatography. The CD analyses confirmed that in all the cases the residues substitutions did not compromise the stability of the protein domain and that the protein adopted the native fold state. (**B**) Nano ES MS data shows that the double mutants BUBR1 L128A-L131A and Y141A-L142A have a very low affinity for the Blinkin mimic peptide compared to the WT protein. Peptide-free BUBR1 is show for comparison. (**C**) 1d ^{1}H NMR spectra of BUBR1 wild-type and binding-site mutants (only an expansion of the up-field methyl region is shown here). Akin to the wild-type, both double-mutants exhibit ring-current shifted methyl signals indicative of tertiary structure. Some differences in chemical shifts presumably arise from Leu-Ala mutation of L128 and L142, which resonate in this region, and potentially subtle local conformational differences/loss of the Y141 ring-current in this area of the structure. (**D**) yeast-two hybrid experiments showed not interaction between the double mutants BUBR1 L128A-L131A and Y141A-L142A and Blinkin. Mated cells were selected in quadruple dropout (SD/-Ade/-His/-Leu/-Trp) plates containing alpha-galactosidase using protocols described in materials and methods.

Supplementary Movies 1-4. Time-lapse microscopy of BUBR1 mutants.

Movie 1. SAC function in a 3FLAG-Venus-BUBR1(WT)-expressing cell as monitored by time-lapse microscopy. The time between one frame and the next is 6 minutes. The time from the NEBD to anaphase for WT BUBR1 was 258 min.

Movie 2. SAC function in a 3FLAG-Venus-BUBR1(KEN26AAA)mutant-expressing cell as monitored by time-lapse microscopy. The time between one frame and the next is 6 minutes. The time from the NEBD to anaphase of BUBR1(KEN26AAA) was 18 min.

Movie 3. SAC function in a 3FLAG-Venus-BUBR1(L128A/L131A)mutant-expressing cell as monitored by time-lapse microscopy. The time between one frame and the next is 6 minutes. The time from the NEBD to anaphase of BUBR1(L128A/L131A) was 180 min.

Movies 4. SAC function in a 3FLAG-Venus-BUBR1(Y141A/L142A)mutant-expressing cell as monitored by time-lapse microscopy. The time between one frame and the next is 6 minutes. The time from the NEBD to anaphase of BUBR1(Y141A/L142A) was 78 min.

Supplemental Experimental Procedures

Y2H Analysis—Human sequences of full length *BUBR1* (*BUBR1*$_{FL}$), *BUBR1*$_{57\text{-}220}$ and *BUBR1*$_{57\text{-}220}$ (E211A-E212A-E213A-E214A), (L128A-L131A), (Y141A-L142A) mutants and multiple fragments encompassing N-terminal Blinkin, including *Blinkin*$_{1\text{-}800}$, *Blinkin*$_{1\text{-}530}$, *Blinkin*$_{531\text{-}800}$, *Blinkin*$_{1\text{-}199}$, *Blinkin*$_{200\text{-}400}$, *Blinkin*$_{300\text{-}400}$, *Blinkin*$_{251\text{-}300}$, *Blinkin*$_{210\text{-}300}$, *Blinkin*$_{225\text{-}250}$, *Blinkin*$_{225\text{-}260}$, *Blinkin*$_{227\text{-}250}$, *Blinkin*$_{205\text{-}250}$, *Blinkin*$_{208\text{-}226}$, *Blinkin*$_{210\text{-}240}$, *Blinkin*$_{210\text{-}226}$ and *Blinkin*$_{208\text{-}226}$ (K212A-K220A-R221A-K223A-K226A) mutant were cloned into pGBKT7 and pGADT7 and fused to GAL4 DNA-binding domains and GAL4 activator domains (Clontech). Y2H protocols were based on the Matchmaker 3 Y2H system (Clontech). Using a PEG/ssDNA/LiAc procedure, pGBKT7 vectors were transformed into the Y187 strain and pGADT7 vectors were transformed into the GOLD strain. For mating, single colonies of Y187[pGBKT7-bait] and GOLD[pGADT7-target] were re-suspended in 0.5 ml 2x YPDA and incubated at 30 °C, 50 rpm for 24 h. Cultures were diluted 1 in 10 with 0.5x YPDA and 100 µl plated on SD/-Leu/-Trp (double dropout) and SD/-Ade/-His/-Leu/-Trp (quadruple dropout) plates containing 1 mg/ml X-α-Gal (Clontech). Plates were incubated at 30 °C and growth monitored for two, three or five days. Shown plates are representative of at least three replicates performed using independent yeast transformants.

Mass Spectrometry— For the preparation of protein-peptide complexes, pure BUBR1$_{57\text{-}220}$ was mixed with an excess of peptide (typically 1:3 molar ratio). After 4 °C incubation of the mixture for few hours, each protein-peptide complex was purified by size exclusion chromatography in a Superdex 75 26/60 column previously equilibrated in 50 mM sodium phosphates buffer pH 6.0 containing 200 mM NaCl. Protein-peptide complex was concentrated to 9 mg/ml and flash frozen before storage at -80 °C. Samples for nano-

electrospray mass spectrometry (Nano-ESI MS) were prepared by dilution of the original 220 μM stock solution to a final concentration of 45 μM and buffer exchanged to 0.2 M ammonium acetate pH 6.9 using Micro biospin chromatography columns (BioRad). High resolution mass spectroscopy measurements were recorded using a Waters LCT Premier mass spectrometer optimized for the transmission of noncovalent complexes. Typically, 3 μL solution containing BUBR1-peptides were electrosprayed from gold-coated glass capillaries. The pressures and accelerating potentials in the mass spectrometer were optimized to remove adducts while preserving non-covalent interactions. The optimum experimental conditions were obtained with a cone voltage of 87 V, capillary voltage of 1.9 kV, ion energy, 80 V; source pressure 2.7 mbar and time-of-flight analyser pressure 3.3e-7 mbar. All spectra were calibrated internally using a solution of cesium iodide (100 mg/mL). Data were processed withMassLynx 4.0 software (Waters/Micromass) with minimal smoothing and without background subtraction.

Protein expression and crystallization— $BUBR1_{57-220}$ and $BUBR1_{57-220}$-$Blinkin_{208-226}$ fusion were expressed in *E. coli* BL21(DE3) Rosetta 2 cells and 2xTY broth while $BUBR1_{57-220}$ and $BUBR1_{57-220}$-$Blinkin_{208-226}$ fusion for NMR studies were expressed in *E. coli* BL21(DE3) using M9 medium containing 0.1% $^{15}NH_4Cl$, ^{13}Glucose and 100 μg/ml ampicillin. Chimeric $BUBR1_{57-220}$-$Blinkin_{208-226}$ was designed by fusion of the *Blinkin* gene fragment encoding for residues S208-K226 to the 5′ end of the *Bubr1* fragment encoding for $BUBR1_{57-220}$ using a flexible linker $(aca/g\text{-}ggc/g\text{-}agt/c)_4$ and expressed as a GST fusion. Cells were grown for 3 h at 25 °C following induction with 0.8 mM IPTG, suspended in 20 mM Tris-HCl pH 8.0, 300 mM NaCl, 1 mM 1,4-dithiothreitol with protease inhibitor cocktail (Roche) and lysed by sonication. All purification steps were identical as those described above.

Small, single crystals were obtained after optimization of two different crystallization conditions derived from sparse matrix screenings (0.1M magnesium acetate-0.1M sodium cacodylate pH 6.0, 15% PEG 6000 and 0.1M MES pH 6.5, 25% PEG 6000) using conventional vapour diffusion methods.

Circular Dichroism— Far-UV circular dichroism spectra were recorded on an AVIV 62-S spectropolarimeter (AVIV, New Jersey, USA) previously calibrated with camphorosulfonic acid and equipped with a temperature control unit. In all experiments, spectra were recorded at 20 °C in a 0.1 cm quartz cell using an average time of 1.0 s, a step size of 0.5 nm, 1-nm bandwidth, and averaged over 20 scans. The dependence of CD signal on protein concentration was calculated by triplicate using independent samples of concentrations ranging between 50 μg/ml to 600 μg/ml (i.e., ≈2-22 μM). For peptide titration experiments pure $BubR1_{57-220}$ was used at concentration 400 μg/ml. After

subtraction of the buffer baseline, the mean molar ellipticity was calculated. For studies on protein stability as a function of temperature, five unfolding curves were recorded upon heating from 20 to 85 °C at a rate of 1°C/min, and 80 s accumulation time. The apparent melting temperature, Tm, was determined from differential melting curves of the function d[θ222](T)/dt. The concentration of protein solutions was determined from amino acid composition analysis at the PNAC facility (Department of Biochemistry, University of Cambridge). All the peptides were N-acetylated.

Protein Analysis—MALDI-TOF, amino acid composition analysis and N-terminal sequencing by the Edman degradation method were conducted at the PNAC facility (Department of Biochemistry, University of Cambridge).

Peptide synthesis—Custom peptides described in this study were synthesized by conventional solid phase methods (Designer Bioscience, Cambridge, UK). Peptide sequences are as follow: SSENKIDFNDFIKRLKTGK; FNDFIKRL; FNDAIKRL; IDFNDFIAALKTGK; KIDFNDFIKRLKTGK; KIDFNDFIKRLKT; IDFNDFIKRLKT; KIDFNDFIKRL; IDFNDFIAALKTGK; IDFNDFIKRLATGKA; DFNDFIKRLKTGK; AIDFNDFIKRLKTGK; KADFNDFIKRLKTGK; KADFNDFIKRLKT; KIDFNDFIKRLATAK; AIAFNDFIKRLATAK; KIDANDFAKRL; KIDANDAIKRL and KADANDAIKRL. Peptides were N-acetylated, desalted and their purity (≥ 95%) confirmed by HPLC/LCMS and MALDI-TOF analyses.

X-ray Diffraction Data Collection and Structure Solution — Prior to the data collection experiments, all crystals were stabilized by gradual transfer to the cryoprotectant solutions containing glycerol and the crystallisation mother liquor. The final cryoprotectant solution contained 35% (v/v) glycerol. Crystals were flash frozen in liquid nitrogen. All X-ray diffraction data collection experiments were performed on in-house PROTEUM X8 (Bruker AXS) X-ray diffraction system at 100K temperature. The cryogenic conditions were maintained by COBRA (Oxford Cryosystems Ltd), a liquidless low temperature device with dedicated nitrogen generator. Crystals diffracted to 2.2 Å resolution. Both crystal forms belonged to space group C2 and contained two molecules of the BUBR1-Blinkin fusion complex in the asymmetric unit resulting in around 56% solvent content. All diffraction data were indexed, scaled and merged using PROTEUM2 software package (PROTEUM2 User Manual, Bruker AXS, 2010).
The crystal structure solution was obtained by molecular replacement method using structure of BUBR1 (PDB 2WVI) as the search probe. Crystallographic refinement was performed in REFMAC5 (Murshudov et al., 1997). The model was improved by several rounds of refinement with REFMAC5 and manual rebuilding using COOT (Emsley and

Cowtan, 2004) resulting in the final *R* factor of 19.3% and *R*free = 24.7% (**Table I**). Electron density maps obtained clearly showed all α-helices and most of the connecting loops were readily traced. Ramachandran plot analysis using PROCHECK (Laskowski et al., 1993) shows that 98.8% residues fall in the preferred and allowed regions, and only 3 residues are present in disallowed regions.

NMR experiments — NMR experiments were performed on samples of ~200 μM uniformly ^{15}N, ^{13}C-labelled protein in 50 mM pH 7.0 sodium phosphate buffer, 200 mM NaCl at 30 ^{o}C (free BUBR1 titration) and 40 ^{o}C (assignment and dynamics of BUBR1-Blinkin complex and chimera). Data were collected on Bruker AvanceIII (600 MHz) and AvanceII (800 MHz) spectrometers equipped with cryoprobes, controlled by Topspin3 (Bruker Biospin Ltd). Backbone atom assignments were completed with standard experiments (Sattler et al., 1999) for the BUBR1-Blinkin complex and extended using 33% sparse, non-uniformly sampled (NUS) experiments and a conventionally-sampled ^{15}N NOESY-HSQC for the chimera. Data were processed using NMRPipe (Delaglio et al., 1995), with reconstruction of NUS time-domain data using MDD1.6 (Jaravine et al., 2006), and analysed in NMRView (One Moon Scientific). Assignment was aided by an NMRView module (Marchant et al., 2008), which provided rapid input for MARS automated assignment (Jung and Zweckstetter, 2004). Titration of BUBR1 incrementally up to 2 moles of Blinkin peptide to one mole of protein was monitored using 2D ^{1}H-^{15}N SOFAST-HMQC spectra (Schanda et al., 2005). Shift changes were ranked based on the weighted, combined ^{1}H and ^{15}N shifts as follows: $\Delta\delta\,(\text{ppm}) = 5\text{x}\sqrt{}[\delta\Delta_{(1H)}]^2 + \sqrt{}[\delta\Delta_{(15N)}]^2$. ^{1}H-^{15}N heteronuclear NOE measurements were performed using a 5 s recycle time or ^{1}H saturation period, run in an interleaved manner.

Stable HeLa cell lines, RNAi and time-lapse microscopy- *BubR1* was cloned into a modified form of pcDNA5/FRT/TO creating pcDNA5/FRT/TO 3FLAG-Venus-*BubR1* and made siRNA resistant to the RNAi oligo (5'GAUGGUGAAUUGUGGAAUAdTdT) (Sigma) by introducing silent mutations. The introduction of mutants into this construct was performed by quick change PCR using Pfu ultra polymerase (Stratagene) and all constructs were confirmed by DNA sequencing. Stable HeLa/FRT/Trex cell lines were generated as previously described (Nilsson et al., 2008). Cells were seeded into 8 well ibiTreat dishes (ibidi) and synchronized with 2.5 mM Thymidine and depleted of endogenous BUBR1 using RNAi Max (Invitrogen) and 100 nM RNAi oligo. Cells were filmed in Leibovitz's L15 medium (GIBCO) on a Deltavision Core microscope (Applied Precision) using a UApo 40X/1.35 NA objective and analyzed using Softworx software.

Immunoprecipitation of BUBR1

Stable HeLa/FRT/TRex cell lines were synchronized with 2.5 mM thymidine and 10 μM MG132 as outlined in **Fig. 4A**. Cells were harvested by mitotic shake off and washed with ice-cold PBS. Cellular pellets were resuspended and lysed for 30 min on ice in lysis buffer (140 mM NaCl, 30 mM Hepes-KOH pH 7.8, 1 mM DTT, 0.2 μM microcystin, 1x protease inhibitor cocktail (Roche), 1x phosphatase inhibitor cocktail (Roche) and clarified by centrifugation at 20,000 rcf at 4°C for 15 min. 1 mg of cell extracts were incubated with 20 μl of GFP-Trap® A beads (ChromoTek) with gentle agitation at 4°C for 30 min. Beads were washed 3 times with ice-cold lysis buffer, resuspended in 2x SDS-sample buffer and boiled 5 min to dissociate the complexes from the beads. The following antibodies were used for western blot analysis at the indicated dilutions: BUBR1 A300-995A (rabbit, Bethyl) 1:500, Blinkin A300-805A (rabbit, Bethyl) 1:500, Cdc20 E-7 (mouse, Santa Cruz Biotechnology) 1:500, Bub3 clone 31 (mouse, BD Transduction Laboratories) 1:500, Mad2 1:500. The non-parametric Mann-Whitny test was used for the statistical analysis of time-lapse experiments. The test was carried out using a two-tail P value with confidence intervals of 95% and performed with the Prism 4 (GraphPad software).

Supplemental References

Delaglio, F., *et al*., (1995). NMRPipe: a multidimensional spectral processing system based on UNIX pipes. J. Biomol. NMR *6*, 277-293.

Emsley, P., and Cowtan, K. (2004). Coot: model-building tools for molecular graphics. Acta Crystallogr. D Biol. Crystallogr*.* *60*, 2126-2132.

Gouet, P., Courcelle, E., Stuart, D.I., and Metoz, F. (1999). ESPript: multiple sequence alignments in PostScript. Bioinformatic*s* *15*, 305-308.

Jaravine, V., Ibraghimov, I., Orekhov, V.Y. (2006). Removal of a time barrier for high-resolution multi-dimensional NMR spectroscopy. Nature Methods *3*, 605-607.

Jung, Y-S., and Zweckstetter, M. (2004). Mars - robust automatic backbone assignment of proteins. J. Biomol. NMR *30*, 11-23.

Marchant, J., Sawmynaden, K., Saouros, S., Simpson, P., and Matthews, S. (2008). Complete resonance assignment of the first and second apple domains of MIC4 from *Toxoplasma gondii*, using a new NMRView-based assignment aid. J. Biomol. NMR assign*.* *2*, 119-121.

Murshudov, G.N., Vagin, A.A. & Dodson, E.J. (1997). Refinement of Macromolecular Structures by the Maximum-Likelihood Method. Acta Cryst. D Biol. Crystallogr*.* *53*, 240-255.

Sattler, M., Schleucher, J., and Griesinger, C. (1999). Heteronuclear multidimensional NMR experiments for the structure determination of proteins in solution employing pulsed field gradients. Prog. Nucl. Magn. Reson. Spectrosc. *34*, 93-158.

Schanda, P., Kupcě, E., and Brutscher, B. (2005). SOFAST-HMQC experiments for recording two-dimensional heteronuclear correlation spectra of proteins within a few seconds. J. Biomol. NMR *33*, 199-211.
