# Supplementary figures and images for: Structure of a Blinkin-BUBR1 Complex Reveals an Interaction Crucial for Kinetochore-Mitotic Checkpoint Regulation via an Unanticipated Binding Site
Source: Structure. 2011 Nov 9;19(11-2):1691–700. doi: 10.1016/j.str.2011.09.017 (PMC3267040; doi:10.1016/j.str.2011.09.017)

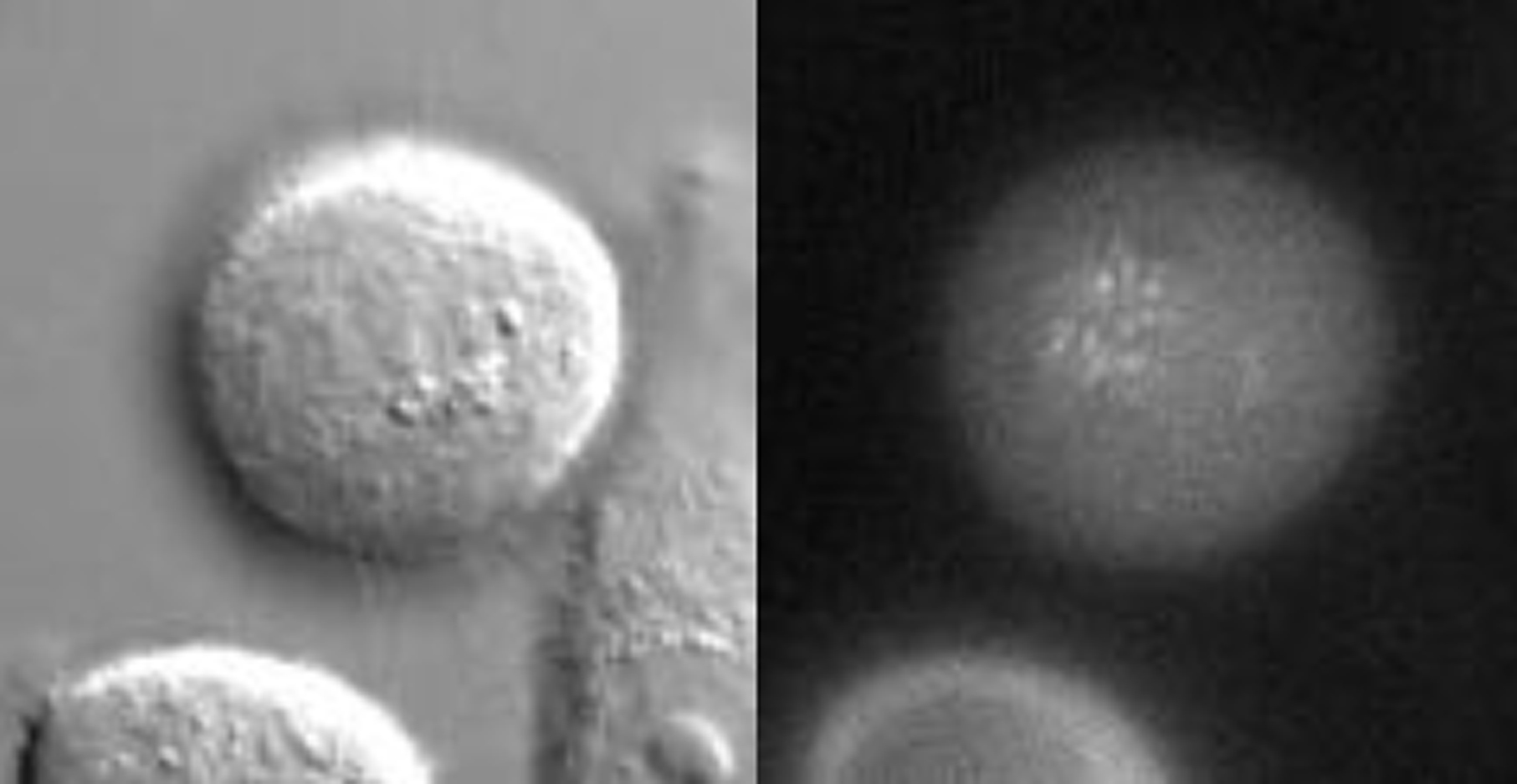

Supplement: Movie S1. Data of cells expressing native BUBR1 protein [file mmc2.jpg]

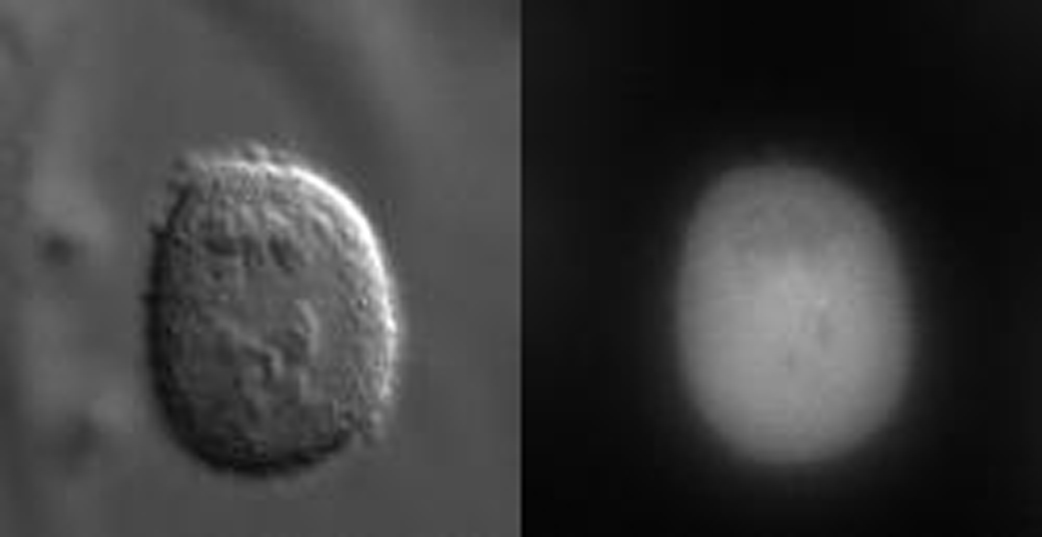

Supplement: Movie S2. Data of cell expressing the BUBR1 mutant (KEN26AAA) [file mmc3.jpg]

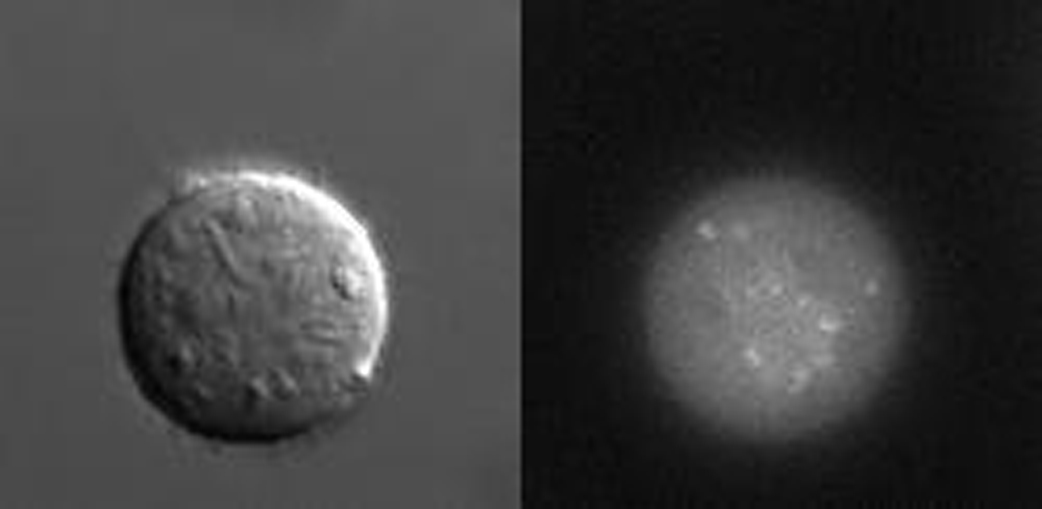

Supplement: Movie S3. Data of cell expressing the BUBR1 double mutant (L128A/L131A) [file mmc4.jpg]

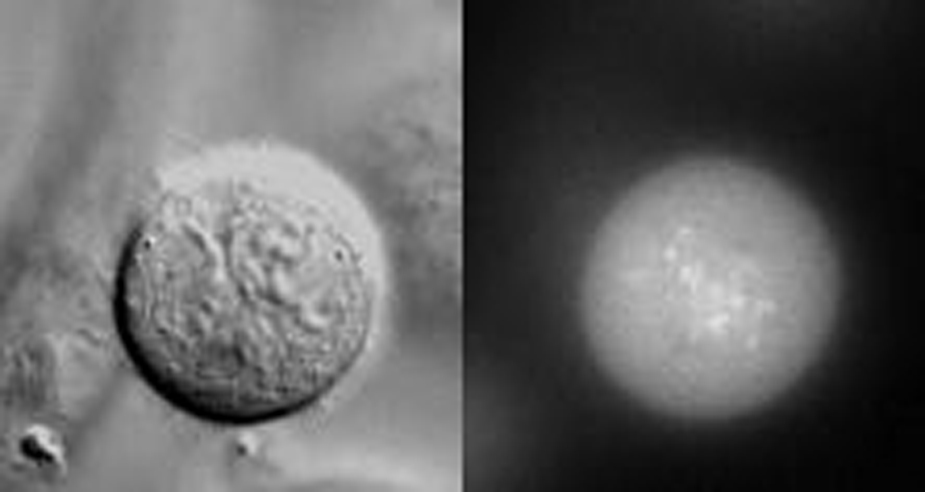

Supplement: Movie S4. Data of cell expressing the BUBR1 double mutant (Y141A/L142A) [file mmc5.jpg]
